# Targeting TRAF3IP2 inhibits angiogenesis in glioblastoma

**DOI:** 10.3389/fonc.2022.893820

**Published:** 2022-08-15

**Authors:** Amin Izadpanah, Fatemeh Daneshimehr, Kurtis Willingham, Zahra Barabadi, Stephen E. Braun, Aaron Dumont, Ricardo Mostany, Bysani Chandrasekar, Eckhard U. Alt, Reza Izadpanah

**Affiliations:** ^1^ Applied Stem Cell Laboratory, Medicine/Heart and Vascular Institute, Tulane University School of Medicine, New Orleans, LA, United States; ^2^ Department of Surgery, Tulane University School of Medicine, New Orleans, LA, United States; ^3^ Division of Regenerative Medicine, Tulane National Primate Research Center, Covington, LA, United States; ^4^ Department of Neurosurgery, Tulane University School of Medicine, New Orleans, LA, United States; ^5^ Department of Pharmacology, Tulane University School of Medicine, New Orleans, LA, United States; ^6^ Department of Medicine, University of Missouri School of Medicine and Harry S. Truman Veterans Memorial Hospital, Columbia, MO, United States; ^7^ Department of Medicine, Isarklinikum Munich, Munich, Germany

**Keywords:** TRAF3IP2, Glioblastoma multiforme, tumor microenvironment, angiogenesis, inflammation

## Abstract

Increased vascularization, also known as neoangiogenesis, plays a major role in many cancers, including glioblastoma multiforme (GBM), by contributing to their aggressive growth and metastasis. Although anti-angiogenic therapies provide some clinical improvement, they fail to significantly improve the overall survival of GBM patients. Since various pro-angiogenic mediators drive GBM, we hypothesized that identifying targetable genes that broadly inhibit multiple pro-angiogenic mediators will significantly promote favorable outcomes. Here, we identified TRAF3IP2 (TRAF3-interacting protein 2) as a critical regulator of angiogenesis in GBM. We demonstrated that knockdown of TRAF3IP2 in an intracranial model of GBM significantly reduces vascularization. Targeting TRAF3IP2 significantly downregulated VEGF, IL6, ANGPT2, IL8, FZGF2, PGF, IL1β, EGF, PDGFRB, and VEGFR2 expression in residual tumors. Our data also indicate that exogenous addition of VEGF partially restores angiogenesis by TRAF3IP2-silenced cells, suggesting that TRAF3IP2 promotes angiogenesis through VEGF- and non-VEGF-dependent mechanisms. These results indicate the anti-angiogenic and anti-tumorigenic potential of targeting TRAF3IP2 in GBM, a deadly cancer with limited treatment options.

## Introduction

Glioblastoma multiforme (GBM) is a grade IV glioma, characterized by aggressive vascularization and intracranial dissemination (1). Clinically, GBM is the most aggressive primary brain tumor with a median survival rate of 14 months despite maximal safe resection and adjuvant chemotherapy (2). A high level of vascularization is a hallmark of GBM. The standard of care, including treatment with temozolomide (TMZ), an alkylating agent that forms the backbone of GBM treatment, is met with a limitation of 50% chemoresistance rate (3). To overcome this clinical challenge, more recently, anti-angiogenic approaches have been used clinically, including the administration of bevacizumab (an anti-vascular endothelial growth factor (VEGF) antibody), cilengitide (an integrin inhibitor), cediranib (an anti-VEGF receptor tyrosine kinase inhibitor), and enzastaurin (a protein kinase C beta inhibitor). Despite some evidence of extending progression-free survival (PFS) and clinical improvement, these anti-angiogenic therapies by themselves failed to improve overall survival (OS) in GBM (4, 5). Therefore, it is critical to identify newer molecules with broader anti-angiogenic effects.

As mentioned above, GBM is characterized by a robust vascular network (6). In fact, the systemic levels of pro-angiogenic mediators such as VEGF, FGF-2, IL8, IL2, and GM-CSF have been shown to be twofold higher in GBM patients. Moreover, GBM is associated with a threefold increase in IL6, IL1β, and TNF-α, reflecting a rich pro-angiogenic environment (6). Several of these pro-angiogenic mediators drive multiple vascular processes, including sprouting angiogenesis, vasculogenesis involving endothelial precursor cell (EPC) recruitment, vasculogenic mimicry involving tumor cells lining blood vessels, and intussusceptive angiogenesis (7, 8). This pathologic angiogenesis, also known as neoangiogenesis, is characterized by enlarged and unorganized vessels with abnormal basement membrane and low pericyte density. Moreover, cancer cell-secreted pro-inflammatory cytokines and chemokines also increase endothelial permeability, yielding leaky vessels resulting in interstitial edema, pressure and intravascular metastasis and dissemination, and ultimately death (9). Of note, in addition to inhibiting neoangiogenesis, anti-VEGF therapy has also been shown to reduce vasogenic cerebral edema in brain tumors (10).

Rapidly progressing tumors exhibit a hypoxic microenvironment, especially in the central tumor core. In fact, pseudopalisading necrosis, a histological hallmark of GBM, reflects progression of the core to ischemic necrosis. The pseudopalisading cells are bordered by microvascular hyperplasia, largely driven by hypoxia-induced HIF-1α activation (11). Interestingly, independent of microenvironmental hypoxia, GBM is predisposed to HIF-1α activation due to *EGFR* (epidermal growth factor receptor) gene amplification and EGFR-dependent PI3K/AKT/mTOR signaling (11). In p53-mutant or p53-deleted GBM, lack of p53 promotes MDM2 ubiquitination and HIF-1α degradation, leading to increased HIF-1α expression and angiogenesis (11). Also, inflammatory cytokines promote angiogenesis, a mechanism amenable to treatment with monoclonal antibodies and small-molecule inhibitors. For example, IL1β induces VEGF expression through p38MAPK- and JNK-dependent AP-1 and NF-κB activation (12). IL6 promotes angiogenesis through the JAK2/STAT3 pathway (13). IL8 induces angiogenesis through JAK2/STAT3 and PI3K/Akt pathways. Of note, PI3K has been shown to independently activate STAT3 (14).

IL-17A, a member of the unique IL-17 cytokine family, plays a causal role in tumor biology, including colorectal cancer, lung cancer, pancreatic cancer, and breast cancer (15). In addition to its role in angiogenesis, IL-17 also exerts pleiotropic pro-tumorigenic effects in GBM. It is prominent in the tumor microenvironment (TME) and significantly overexpressed in GBM (16). In GBM cells, IL-17 promotes cell migration and invasion, with concomitant increases in PI3K, AKT, MMP2/9, Twist, and Bmi1 and reduced expression of the tight-junction protein ZO-1 (16). In contrast, IL-17 has been shown to contribute to the formation of an immunosuppressive TME *via* decreasing CD8+ T cells and increasing myeloid-derived suppressor cells (17). Consistently, inhibiting IL-17 increases the anti-tumorigenic potential of tumor-infiltrating lymphocytes (18). The synergistic anti-tumorigenic effects of immunotherapy and anti-angiogenic therapy are well recognized (19). Developing strategies and targets that exert anti-angiogenic and immunotherapeutic effects are a major clinical goal.

IL-17 signals predominantly *via* the IL-17R complex that activates inflammatory signaling. For example, binding of IL-17A to the IL17RA/RC complex recruits TRAF3IP2 (also known as CIKS or ACT1) *via* a homotypic interaction mediated by the SEFIR domain, resulting in activation of multiple downstream signaling cascades. TRAF3IP2 exerts a U-box E3 ubiquitin ligase activity to polyubiquinate TRAF6 at K63, recruitment of TAK1, activation of IKK, IkBa phosphorylation/degradation, and NF-kB activation. TRAF6 also activates MAPK and c/EBP. By associating with the EGFR and IL-17R complex, TRAF3IP2 also activates ERK5 (20, 21). It also plays a role in mRNA stability of some chemokines, including CXCL1. Interestingly, both IKK and TBK1 phosphorylate TRAF3IP2 at serine residue 331, leading to the formation of the TRAF3IP2/TRAF2/TRAF5/ASF complex, which prevents ASF binding to the 3′ UTR of CXCL1 mRNA and CXCL1 mRNA degradation. Interestingly, CXCL1 is a potent driver of angiogenesis (22), and its increased expression predicts poor outcomes in GBM (23). These signaling interactions, downstream of IL-17/IL-17R, also activate stress-activated kinases and pro-angiogenic transcription factors, including p38MAPK and AP-1, which regulate the expression of VEGF and ANG2. We have previously shown that TRAF3IP2 is expressed at significantly higher levels in malignant U87 and U118 glioblastoma cells compared to the non-malignant glial cell line SVG p12 (24). We have also demonstrated that targeting TRAF3IP2 reduces VEGF expression, likely through blockade of NF-κB signaling, in a flank model of GBM (24). Indeed, NF-κB activation is a critical regulator of VEGF expression in glioblastoma (25). Here, we extend our findings to an intracranial model of GBM to accurately recapitulate neoangiogenesis in the brain tumor microenvironment. Because of the comprehensive function and upstream position of TRAF3IP2, we tested the hypothesis that targeting TRAF3IP2 decreases neoangiogenesis.

## Results

### Detection of high levels of TRAF3IP2 in the GBM tumor microenvironment

**Figure 1:**
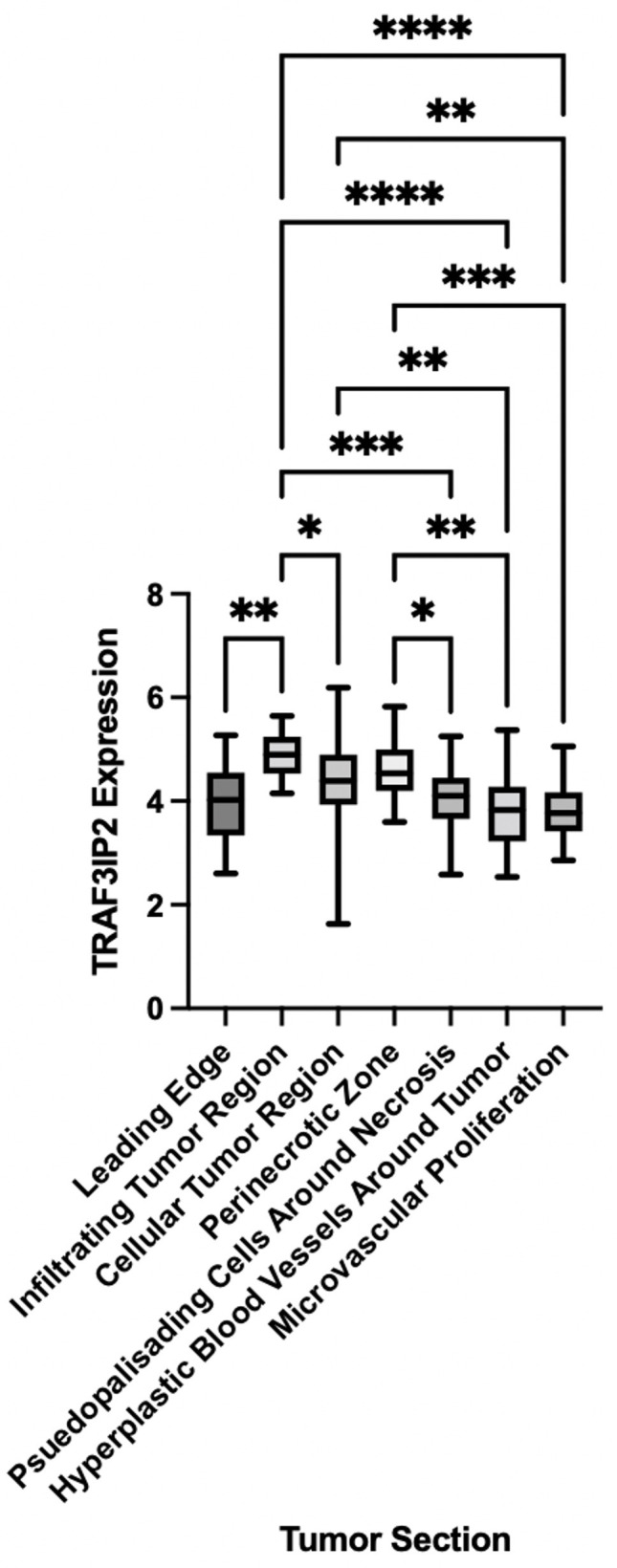
TRAF3IP2 is expressed at the highest levels at the infiltrating edge of the tumor. RNA sequencing data were mined from the IVY GBM Atlas project, accessed through https://glioblastoma.alleninstitute.org. Significance was calculated by ANOVA: *p < 0.05, **p < 0.01, ***p < 0.001, ****p < 0.0001.

Analysis of RNA sequencing data from the IVY GBM Atlas project, accessed through https://glioblastoma.alleninstitute.org, demonstrated a significant elevation in TRAF3IP2 expression in vascularized areas of the GBM TME such as the tumor-infiltrating region, suggesting the causal role of increased TRAF3IP2 in GBM angiogenesis (**Figure 1**). The levels of TRAF3IP2 in GBM are consistently higher, relative to normal brain as reported by us previously (24) and as shown in **Figure 4**. The difference in TRAF3IP2 expression between normal brain and GBM is likely greater than the difference in TRAF3IP2 expression between various tumor tissues. The tumor-infiltrating region is principally involved in the metastatic cascade that drives GBM dissemination, and the region itself is a major site of neo-angiogenesis and microvascular proliferation that promotes new vessel growth and tumor cell spread. Therefore, the observation that the tumor-infiltrating region of GBM has the highest expression of TRAF3IP2 is a statistically and translationally significant finding that points to TRAF3IP2 as a key driver of metastasis, which depends on angiogenesis at the infiltrating edge of the tumor.

### Targeting TRAF3IP2 significantly reduces angiogenesis in GBM

**Figure 2:**
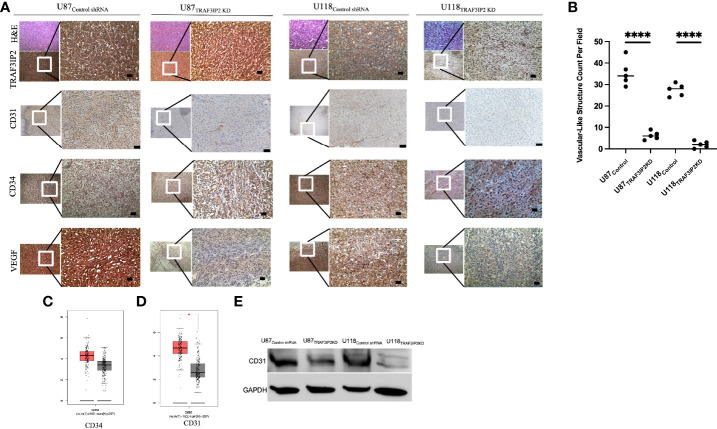
Immunohistochemical analysis demonstrating that knockdown of TRAF3IP2 in GBM reduces angiogenic markers. The U87 and U118 cells were transduced with anti-TRAF3IP2-shRNA (U87_TRAF3IP2KD_ and U118_TRAF3IP2KD_, respectively) to silence TRAF3IP2 or Scrambled-shRNA (U87_Control shRNA_ and U118_Control shRNA_, respectively) as a control. The cells (U87_TRAF3IP2KD_ or U87_control shRNA_ or U118_TRAF3IP2KD_ or U118_Control shRNA_) were used to induce intracranial tumors to generate an orthotopic mouse GBM mouse model. The cells (3 × 10^5^ cells) were injected into the left somatosensory cortex (SSCx) of NSG mice for tumor induction. The animals were euthanized 28 days post-tumor induction. Histology and immunohistochemistry revealed that U87_TRAF3IP2KD_ and U118_TRAF3IP2KD_ compared to control had significantly less TRAF3IP2 expression, which correlated with decreased CD31 and CD34 (endothelial vascular markers) and VEGF. Higher magnification of selected areas is shown (scale bar is 100 μm) **(A)**. Quantification of vascular-like structures **(B)**. Analysis using GEPIA revealed that GBM tumors express higher levels of CD34 **(C)** and significantly higher levels of CD31 (p < 0.05) **(D)**, compared to normal brain controls. Targeting TRAF3IP2 in GBM cell lines decreases protein levels of CD31 **(E)**. *P<0.05, ****P<0.0001.

TRAF3IP2 expression in U87 and U118 cells was silenced/knocked down by lentiviral transduction of specific shRNA (U87_TRAF3IP2KD_ and U118_TRAF3IP2KD_, respectively). Scrambled shRNA served as the control (U87_Control_ and U118_Control_, respectively). Previously, we reported a decreased expression of TRAF3IP2 in U87_TRAF3IP2KD_ and U118_TRAF3IP2KD_ compared to corresponding controls (24). Here, we administered U87_TRAF3IP2KD_, U118_TRAF3IP2KD_, or corresponding controls (U87_control_ and U118_Control_) to induce intracranial tumors to generate an orthotopic mouse GBM model. The cells (3 × 10^5^ cells) were injected into the left somatosensory cortex (SSCx) of immunodeficient NSG (NOD scid gamma) mice for tumor induction and euthanized after 28 days. The data show a significant reduction in tumor formation by both U87_TRAF3IP2KD_ (0.6 ± 0.3 mm^3^) and U118_TRAF3IP2KD_ (0.3 ± 0.2 mm^3^) cells compared to U87_control_ (1.2 ± 0.6 mm^3^) and U118_Control_ (0.9 ± 0.3 mm^3^; p < 0.05). Histological analysis shows a significant reduction in the expression levels of TRAF3IP2, vascular markers CD31 and CD34, and VEGF, as well as decreased vasculature-like structures in U87_TRAF3IP2KD_- or U118_TRAF3IP2KD_-derived residual tumors compared to tumors formed by corresponding controls (**Figure 2A**). Further, Western blot analysis showed significant downregulation in CD31 expression in both U87_TRAF3IP2KD_ and U118_TRAF3IP2KD_ residual tumors compared to their controls (**Figure 2B**).

### *In vitro* angiogenesis

**Figure 3:**
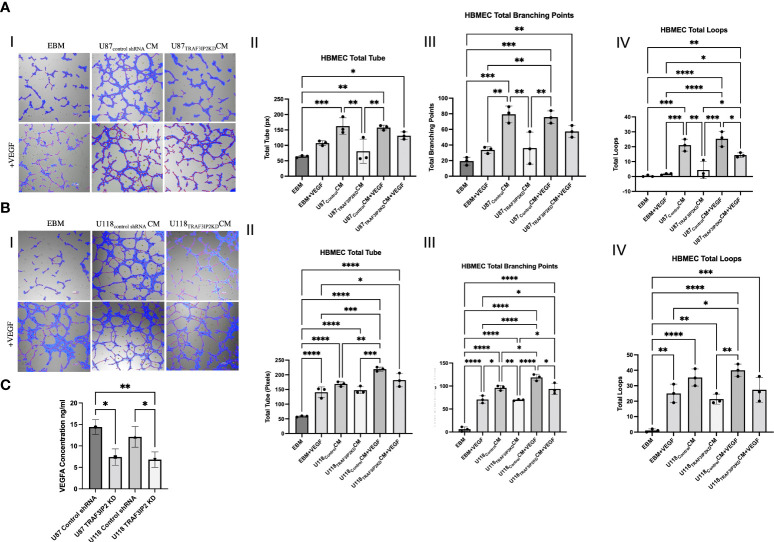
Tube formation assays demonstrating that knockdown of TRAF3IP2 in GBM reduces angiogenesis. HBMEC (human brain microvascular endothelial cells) were cultured in the following conditions: normal media (EBM), conditioned media (CM) from U87_Control shRNA_ or U118 _Control shRNA_ cells, or CM from U87_TRAF3IP2KD_ or U118_TRAF3IP2KD_ cells (Figure 3AI, 3BI). The addition of exogenous VEGF at a concentration of 40 ng/ml was also performed (bottom rows, AI and 3BI). Effects of CM from U87_TRAF3IP2KD_ **(A)** or U118_TRAF3IP2KD_ **(B)** with or without VEGF on angiogenesis parameters including total tube [measured in pixels (px)], total branching points, and total loops are shown (I-IV). U87_TRAF3IP2KD_ and U118_TRAF3IP2KD_ compared to U87_Control shRNA_ and U118_Control shRNA_ secreted significantly lower amounts VEGF in the medium by enzyme-linked immunosorbent assay (ELISA) **(C)**. Significance was calculated by ANOVA: *p < 0.05, **p < 0.01, ***p < 0.001, ****p < 0.0001.

To elucidate the mechanisms by which targeting TRAF3IP2 reduces angiogenesis, an *in vitro* tube formation assay was performed. Human brain microvascular endothelial cells (HBMEC) were cultured in normal media (endothelial basal media or EBM), conditioned media (CM) from U87_Control_ or U118_Control_ cells, or CM from U87_TRAF3IP2KD_ or U118 _TRAF3IP2KD_ cells (**Figure 3AI, 3BI**). Total tube (in pixels), total branching points, and total loops were quantified as indices of the degree of neoangiogenesis and vascularization (**Figure 3**). Results indicate that while treatment with U87_Control_ CM increased total tube (**Figure 3AII**), total branching (**Figure 3AIII**), and total loops compared (**Figure 3AIV**) to control EBM, treatment with U87TRAF3IP2KD CM treatment significantly reduced HBMEC angiogenesis compared to U87_Control_ CM (**Figure 3**). Collectively, these results suggest that U87_Control_ secrete angiogenic factors that accelerate angiogenesis, and silencing TRAF3IP2 inhibit the secretion of angiogenic factors by U87_TRAF3IP2KD_ cells. Since VEGF is a critical driver of angiogenesis and silencing TRAF3IP2 inhibits VEGF expression in GBM (21, 24), we next investigated the effect of exogenous VEGF on tube formation. The results indicate that addition of VEGF (40 ng/ml) to U87_TRAF3IP2KD_CM restored tube formation, and total branching points to similar levels as U87_Control_CM and U87_Control_CM + VEGF (**Figure 3A**). Importantly, total loop formation in U87_TRAF3IP2KD_CM + VEGF was still significantly lower than U87_Control_CM + VEGF (**Figure 3AIV**). This may indicate that the amount of VEGF added did not fully restore the VEGF inhibition induced by TRAF3IP2 silencing. Additionally, while addition of VEGF to U87_TRAF3IP2KD_CM restored tube formation and total branching points to similar levels as U87_Control_CM and U87_Control_CM + VEGF, the mean values in U87_TRAF3IP2KD_CM+VEGF were lower but did not reach statistical significance. Interestingly, U87_Control_CM and U87_Control_CM + VEGF displayed similar levels of total tube formation, total branching points, and total loops, suggesting that the effects were already saturated and the addition of exogenous VEGF had no further detectable effect. Notably, addition of VEGF to EBM, rather than CM, non-significantly increased total tube formation, total branching points, and total loops. These results could also be due to the presence of non-VEGF growth factors in U87_Control_ CM that might be driving angiogenesis. Consistently, the lack of restoration of those non-VEGF factors accounts for the subtotal restoration of angiogenesis in the U87_TRAF3IP2KD_ CM +VEGF group.

Similarly, treatment with U118_Control_ CM significantly increased total tube, total branching, and total loops compared to EBM (**Figure 3B**). U118_TRAF3IP2KD_ CM compared to U118_Control_ CM resulted in decreased total tube formation and total loops that did not reach statistical significance but significantly decreased total branching points (**Figure 3BIII**). Addition of VEGF to U118_TRAF3IP2KD_CM resulted in significantly increased total branching points and a non-significant increase in total loops and total tube (**Figure 3BII–BIV**). Total branch points, total loops and total tube in U118_TRAF3IP2KD_ CM+VEGF were similar to U118_Control_ CM. Further, comparison of U118_TRAF3IP2KD_ CM+VEGF and U118_Control_ CM+VEGF identifies the putative role of non-VEGF factors, especially in total branching points. To confirm that the decreased tube formation is due to lack of VEGF, we measured VEGF levels by ELISA, and results showed a significantly lower concentration of VEGF (~40%) in supernatant of U87_TRAF3IP2KD_ and U118_TRAF3IP2KD_ compared to U87_control shRNA_ and U118_TRAF3IP2KD_ cultures (p < 0.001) (**Figure 3C**).

### Transcriptomic analysis identifies the pro-angiogenic function of TRAF3IP2 in GBM

**Figure 4:**
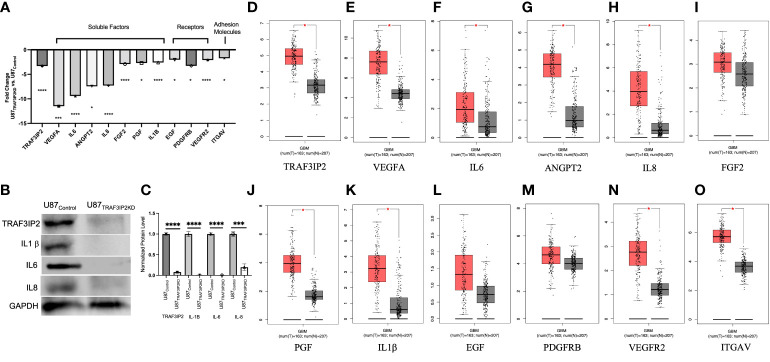
Targeting TRAF3IP2 in GBM decreases pro-angiogenic factors and receptors. Gene expression fold changes (U87_TRAF3IP2KD_ vs. U87_Control shRNA_) are shown. *p < 0.05, ***p < 0.001, ****p < 0.0001 **(A)**. Protein levels of key angiogenic factors in U87_TRAF3IP2KD_ vs. U87_Control_ **(B)** and quantification **(C)**. Levels of key angiogenic factors in GBM vs. normal tissue: clinical GBM tumor samples and data (red bars) are from The Cancer Genome Atlas (TCGA) red bars and normal controls (gray bars) are from Genotype-Tissue Expression (GTEx) accessed through GEPIA (http://gepia.cancer-pku.cn), p < 0.01 **(D–O)**.

**Figure 5:**
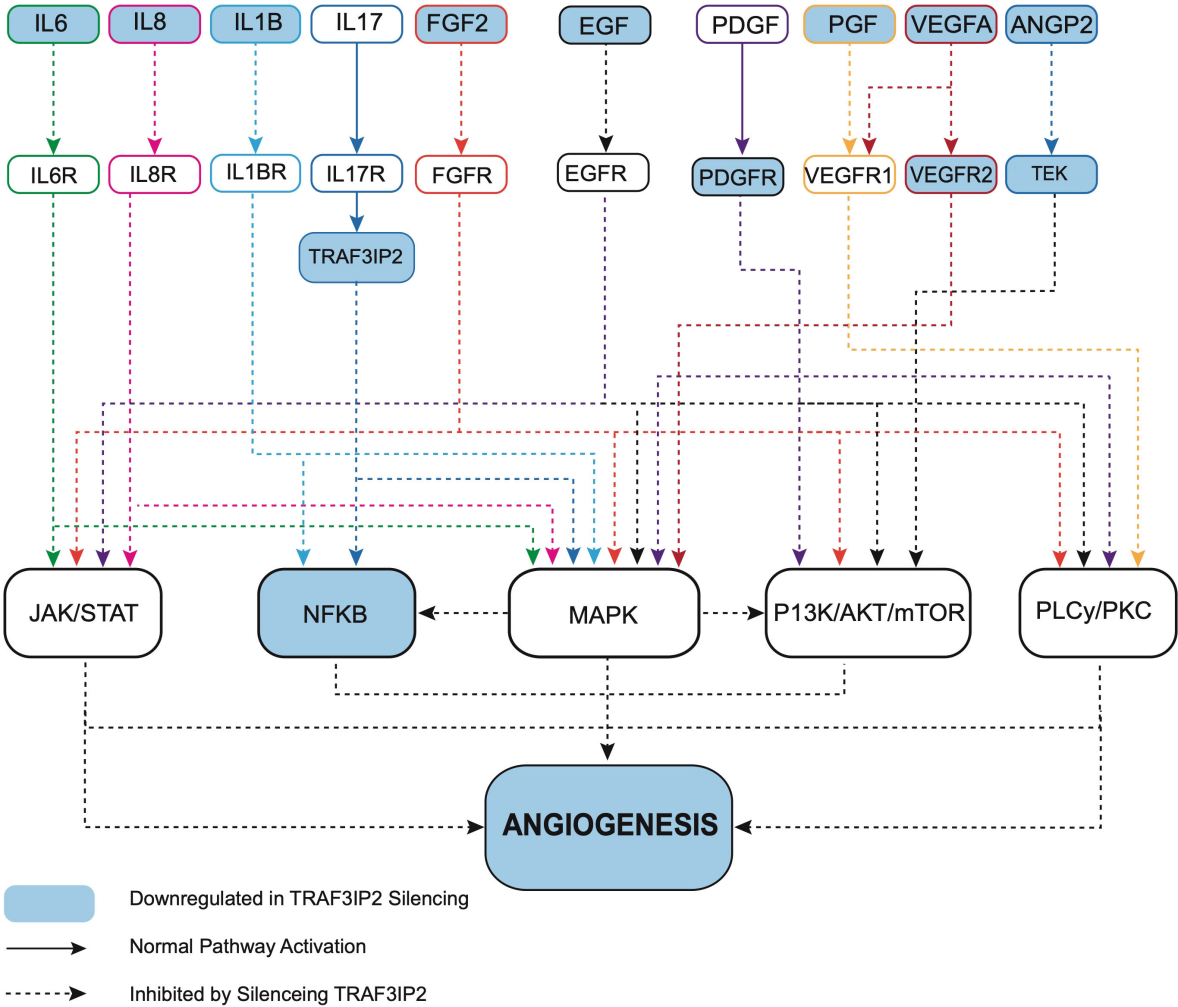
Ingenuity pathway analysis (IPA) reveals that targeting TRAF3IP2 coordinately inhibits multiple pro-angiogenic pathways. IPA reveals that targeting TRAF3IP2 coordinately inhibits multiple pro-angiogenic pathways. Gene expression analysis revealed a differentially expressed gene list between the U87_TRAF3IP2KD_ and U87_control shRNA_ cells. IPA^®^ canonical pathway analysis and molecular network analysis revealed HIF-1α signaling, tumor microenvironment signaling, and neuroinflammation pathways as top regulated pathways.

**Figure 6:**
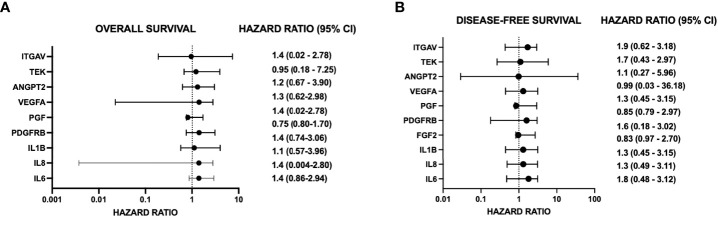
Forest plots demonstrate hazard ratios and 95% confidence intervals (CI) for angiogenic genes significantly changed by targeting TRAF3IP2. Data are from The Cancer Genome Atlas (TCGA), accessed through GEPIA (http://gepia.cancer-pku.cn). Group cutoff for high expression vs. low expression of the gene of interest is the median. Hazard ratio (HR) is calculated based on the COX proportional hazard model. Hazard ratios are for the “high expression” group for each gene and refer to overall survival (OS) Panel **(A)** or disease-free survival (DFS) Panel **(B)**.

Transcriptomic analysis of U87_TRAF3IP2KD_ vs. U87_Control_ reveals perturbation in pro-angiogenic cytokines, growth factors and their receptors, and cell adhesion molecules. For example, targeting TRAF3IP2 inhibited IL1b, IL6, IL8, FGF2, EGF, PDGFRB, PGF, VEGFA, VEGFR2, ANGPT2, and ITGAV expression in GBM cells (**Figure 4**). Western blot analysis showed a significant reduction in IL1β, IL6, and IL8 levels (**Figure 4B**). Using IPA pathway analysis, we then analyzed the angiogenesis pathways that are affected by TRAF3IP2 knockdown. As shown in **Figure 5**, the pathway analysis showed a series of novel findings indicating the effect of silencing TRAF3IP2 in GBM angiogenesis. For example, silencing TRAF3IP2 resulted in a significant reduction in IL1β expression which can result in reduced NFκB and MAPK activation/expression. In addition, silencing TRAF3IP2 inhibited FGF2, which regulates MAPK activation through its receptor FGFR (**Figure 5**). FGF2 regulates the self-renewal of multiple stem cell types. In fact, FGF2 is supplemented in growth medium for *ex vivo* culture of cancer cells, including GBM (26, 27). Silencing TRAF3IP2 also decreased the expression of EGF and PDGFRB, which are involved in metastasis *via* increasing angiogenesis (28). More importantly, silencing TRAF3IP2 inhibited the expression levels of VEGF, VEGFR, and ANGPT2, potent angiogenic markers in GBM (29, 30). Among the cell adhesion molecules, silencing TRAF3IP2 reduced ITGAV (Integrin-αV) expression which is known to be involved in the migration of glioma cells and is thus considered to contribute to invasiveness (31). The expressions of these markers are markedly changed in GBM and are highly associated with disease-free survival and overall survival (**Figure 6**).

## Discussion

For the first time, our novel data show that TRAF3IP2 levels are significantly upregulated in highly vascularized areas in the GBM tumor microenvironment, including the tumor-infiltrating region (**Figure 1**). This strongly implicates TRAF3IP2 as a critical contributor of GBM dissemination in the brain, which depends on angiogenesis-dependent hematogenous routes. Further, data from The Cancer Genome Atlas (TCGA) reveal that human GBM exhibits significantly higher levels of TRAF3IP2 (**Figure 4D**). Importantly, our data demonstrate that silencing TRAF3IP2 significantly reduced the expression of pro-angiogenic markers, including CD31, CD34, and VEGF, in induced intracranial GBM tumors (**Figure 2**). Our functional *in vitro* results corroborate our *in vivo* findings and demonstrate that targeting TRAF3IP2 reduces the ability of GBM to induce brain endothelial cell-mediated vascularization. We showed that this effect is due to a significant reduction in VEGF expression (**Figure 3**). It has been shown that angiogenic factors, such as VEGF, are released directly or *via* exosomes into the GBM tumor microenvironment to enhance angiogenesis (32). Therefore, it is plausible that targeting TRAF3IP2 may affect the direct as well as exosome-mediated release of angiogenic mediators. Our transcriptomic analysis identified that targeting TRAF3IP2 perturbed the expression of several genes with pro-angiogenic effects. Specifically, we identified that silencing TRAF3IP2 reduces the levels of proinflammatory cytokines, growth factors and receptors, and cell adhesion molecules that are involved in angiogenesis. Therefore, silencing TRAF3IP2 has the therapeutic potential in GBM by targeting neoangiogenesis.

In addition to inhibiting the release of pro-angiogenic mediators, our results show that silencing TRAF3IP2 in GBM cells decreases the expression of IL1b, IL6, and IL8, which are proinflammatory and pro-angiogenic (**Figure 4**). It was recently shown that exposure of GBM cells to IL1β significantly changes the proinflammatory secretome, including IL8 and IL6 (33). IL6 promotes GBM development through enhancement of cell invasion and migration (34). Indeed, IL6 signaling through STAT3 is required for glioma development in a preclinical model (35). Increased STAT3 activation, in the form of phosphorylated STAT3, was confirmed in GBM (grade IV), anaplastic astrocytoma (grade III), and diffuse astrocytoma (grade II) (34, 35). Interestingly, STAT3 upregulates VEGF and VEGFR2, thus increasing angiogenic signaling in GBM (34). In addition to pro-angiogenic mediators, STAT3 also upregulates cyclin D1 and Bcl-2, resulting in rapid tumor growth through increased cell-cycle progression and reduced cell death (34). GBM-derived IL8 has also been shown to induce brain endothelial cell permeability and is found at elevated concentrations at the tumor margin of resection, suggesting its role in tumor invasion and dissemination (36, 37). Exposure of human brain microvascular endothelial cells to the U87-CM (U87-conditioned medium demonstrated to contain elevated IL8) induced endothelial remodeling and permeability through ERK. IL8 has also been shown to promote changes in vascular endothelial cadherin (VE-cadherin) localization away from cell junctions, suggesting structural changes that enhance vascular remodeling (36). Further, an association was shown between GBM expression of IL1β, IL6, and IL8 and overall survival and disease-free survival [(hazard ratios for increased expression range from 1.1 to 1.4 (overall survival) and 1.3 to 1.8 (disease-free survival, respectively)] (**Figures 6A, B**) (**Supplementary Figures S1C, D**; **S2G, H**).

Among various growth factors and receptors, we found that targeting TRAF3IP2 decreased EGF, PGF, and PDGFRB expression (**Figure 4**). In GBM, FGF2 enhances tumor growth, angiogenesis, and cancer stem cell renewal. FGFR1, the receptor for FGF, through activation of PLC-γ, leads to resistance to radiation- and hypoxia-induced angiogenesis (26). While the critical role of FGF2 and the therapeutic potential of targeting its activity are recognized preclinically, there is no clinical grade FGF2 antagonist or FGFR modulator. Importantly, blockade of FGF2 by a monoclonal antibody and FGFR1 by RNA interference significantly, but did not completely, arrest GBM growth, through downregulation of MAPK and AKT. However, an improvement in efficacy was observed when anti-FGF therapy was combined with TMZ (38).

In addition to inflammatory mediators with pro-angiogenic effects, our data also show an increased EGF expression in GBM (**Figure 4**). In GBM, EGF/EGFR signaling has been shown to drive metastasis *via* STAT3-mediated NF-κB activation. PI3K/AKT and MAPK pathways are also critical in the induction and activation of matrix metalloproteinase 9 (MMP9) (39). In fact, secreted MMP9 enables the rapid migration and dissemination of GBM both intracranially and extracranially (40). Interestingly, MMP9 expression predicts TMZ response on survival, with increased expression of MMP9 portending decreased survival and resistance to TMZ (41). In addition to cell invasion, EGF/EGFR also drives metastasis through angiogenesis. While the myriad pro-tumorigenic roles of EGF/EGFR are reviewed elsewhere (42), its role in driving angiogenesis makes it an attractive target in blocking GBM dissemination (28).

Our data also show increased PDGFR expression in the brain xenograft model of GBM, and this was confirmed by the clinical GBM tumor sample data from TCGA and GEPIA (**Figure 4**), and this was confirmed by the clinical GBM tumor sample data from TCGA and GEPIA (**Figure 4K**). The PDGF system and its members are also shown to promote GBM growth and metastasis, as well as neoangiogenesis (43). In fact, a histologic analysis of GBM tumors revealed the highest expression of PDGFRB, compared to other members of the PDGF family, especially in regions of hyperplastic blood vessels (43). Similarly, in regions of microvascular proliferation and the leading edge of the tumor, PDGFRB is highly expressed (43). In contrast, PDGFRB showed a decreased expression in the pseudo-palisading regions (43), indicating the critical role of PDGFRB in angiogenesis and GBM invasion, two critical phenotypes that are the subject of therapeutic intervention.

PGF (placental growth factor) is a member of the VEGF family, and its function in normal physiology is poorly understood. In cancer, however, PGF has been shown to drive neoangiogenesis. PGF binds the FLT1 receptor to directly stimulate pro-angiogenic effects. Indirectly, PGF shifts VEGFA binding from FLT1 to VEGFR2, resulting in a significant increase in VEGFA-mediated angiogenesis. In addition to its role in angiogenesis, PGF also plays a role in immunomodulation. PGF has been shown to downregulate type 1 T helper immune responses by modulating dendritic cells (44). The secretion of PGF was found to be significantly increased in the Th17 subset of T helper cells and promoted angiogenesis. PGF also supported the pathogenic differentiation of Th17 cells through STAT3. Similar to IL-6, PGF increased IL-17 production but suppressed the formation of regulatory T cells, leading to autoimmunity in the form of autoimmune encephalomyelitis and collagen-induced arthritis (45). Interestingly, TRAF3IP2 functions as an adaptor protein in canonical IL-17 signaling. Consistently, TRAF3IP2 is recognized to play critical roles in the pathogenesis and progression of various autoimmune diseases (46). We previously demonstrated the role of TRAF3IP2 in GBM for the first time *in a flank model* (24) and here provide novel evidence of its role in driving pathogenic angiogenesis in an *intracranial model of GBM*. This is significant as our intracranial model of GBM recapitulates the human brain tumor microenvironment.

In GBM, specifically, tumor-derived PGF induces the generation of TGFβ+ regulatory B cells, which suppress CD8+ T-cell proliferation and release of perforin and granzyme B (47). Consistent with its role in modulating both immunity and angiogenesis (48), our data show that targeting TRAF3IP2 downregulates PGF expression and signaling (**Figure 4**), suggesting that targeting TRAF3IP2 potentially suppresses both angiogenesis and immune suppression in GBM. Therefore, TRAF3IP2 may be an ideal target to decrease angiogenesis and improve an antitumor immune response in GBM. This is especially relevant since the combination of anti-angiogenic therapy and immune checkpoint blockade, which stimulates antitumor immune response, is suggested to be a more effective in treating malignancy (49).

VEGF is considered the most potent pro-angiogenic cytokine, and drugs, including bevacizumab, a monoclonal antibody against VEGF, have been designed to target its function in GBM (50, 51). However, these drugs have limitations, as evidenced by the eventual progression of the tumor and recurrence (52). In addition to promoting neoangiogenesis in GBM, VEGF has also been shown to promote proliferation of GBM stem-like cells through VEGFR2 (53). Paradoxically, despite a reduction in blood supply and angiogenesis, the anti-VEGF treatment has been shown to increase tumor cell invasion in GBM (29), potentially due to an enhanced hypoxic microenvironment and a feedback activation of HIF-1a and PI3K. Both pathways drive pro-tumorigenic signaling, including metabolic changes favoring glycolysis, which fuels cell division through the pentose phosphate cycle, and increases invasiveness partly through the acidification of the ECM by lactic acid. This critical evidence explains the suboptimal clinical outcomes from anti-VEGF therapy and identifies invasiveness and metabolic remodeling as alternative pathways that GBM activates to resist anti-angiogenesis therapy. Nevertheless, the anti-VEGF-mediated reduction of vascularization is an important element in the treatment of GBM and might be more efficacious as an adjunct to other therapies. For example, VEGF blockade results in a more mature dendritic cell phenotype in the brain and reduces levels of PD-1 (exhaustion marker and suppressor of activity CD8+ T cells) on brain-infiltrating CD8+ T cells, changes that result in improved antitumor immunity (54). This is consistent with the current view that immunotherapy combined with anti-angiogenic therapy might promote better outcomes.

Angiopoietin 2 (ANGPT2) has also been identified as a therapeutic target in GBM (55). ANGPT2 binds to its receptor tyrosine kinase, TEK (Tie2). During inflammation, ANGPT2 *via* TEK leads to increased endothelial permeability (56). In human brain tumors, its expression was found to be markedly elevated, compared to low or undetectable levels in a normal brain (55). ANGPT2 levels also positively correlate with WHO grade of tumors in addition to infiltrating macrophages/monocytes. GBM patients were also found to have increased levels of ANGPT2 preoperatively (55). ANGPT2 is expressed at the site of vascular remodeling, such as tumor vessels in the leading and infiltrating edges, as well as microvascular hyperplastic regions of GBM (57). ANGPT2 has also been postulated to be partly responsible for bevacizumab resistance, suggesting that dual targeting of VEGF and ANGPT2 may be more effective (30). In fact, bevacizumab was shown to upregulate ANGPT2, and blockade of both VEGF and ANGPT2 extended survival, decreased vascular permeability by potentially reducing vasogenic cerebral edema, and favored antitumor immunity by enhancing tumor infiltrating lymphocytes and decreasing tumor-associated macrophages (30).

Our robust mechanistic pathway analysis also demonstrates that silencing TRAF3IP2 regulates the expression of pro-angiogenic mediators (**Figure 5**). These data are supported by clinical tumor data from TCGA (**Figure 4**), which reveal that in human GBM tumors, TRAF3IP2 expression as well as VEGF, IL6, ANGPT2, IL8, PGF, IL1b, ITGAV, and VEGFR2 are significantly upregulated compared to normal tissue. Further, generally, hazard ratios for high expression of such pro-angiogenic genes are >1 for both OS and disease-free survival (DFS) (**Figure 6**; **Supplementary Figure S3**). It is important to note, however, the weakness of looking at bulk survival data, as it does not factor variables which may be confounding, such as age, gender, and, importantly, treatment. While significance was generally not achieved by our analysis (95% confidence intervals), it is important to note the general trend that a higher expression of these genes is associated with both worse OS and DFS. Future analysis should prioritize stratification to avoid confounding, yet also maintain sufficient group sizes to achieve significance.

## Conclusion

Using a brain xenograft model, we demonstrate the critical role of TRAF3IP2 in driving GBM angiogenesis. TRAF3IP2 is expressed at higher levels in GBM tissue and correlates with VEGF expression and vascularity. Our studies further demonstrate that targeting TRAF3IP2 inhibits angiogenesis by reducing the secretion of multiple pro-angiogenic mediators, including VEGF, leading to reduced angiogenesis. This affect was restored sub-maximally by the addition of recombinant VEGF, suggesting that TRAF3IP2 also induces non-VEGF factors that drive angiogenesis. Pathway analysis reveals that targeting TRAF3IP2 significantly reduces the expression of multiple pro-angiogenic cytokines and factors and/or their receptors. Further, TRAF3IP2 itself is a potent activator of NF-κB, MAPK, JNK, and AP-1, all of which induce the expression of pro-angiogenic genes. Due to inhibition of multiple pro-angiogenic mediators, targeting TRAF3IP2 as an adjunctive therapy may potentially increase the efficacy of current anti-GBM treatment regimens with anti-angiogenic components. Future studies will assess the efficacy of targeting TRAF3IP2 as a monotherapy or a combination therapy in robust animal models of heterogeneous primary GBM, with reductions in tumor size, angiogenesis, cerebral edema, and improvements in overall and disease-free survival as critical endpoints.

## Material and methods

### Cell culture

Human malignant GBM cell lines U87 and U118 and primary human brain epithelial cells (HBEC) were purchased from ATCC^®^ (Rockville, MD, USA). The vendor authenticated all cell lines for sterility (mycoplasma, aerobic and anaerobic) and declared free of pathogens (PCR-based assay for HIV, HepB, HPV, EBV, and CMV). U87 and U118 cells display a relatively high colony forming efficiency on agarose, indicating their transformation and tumorigenic potential. We monitored their tumorigenic potential *in vivo*. The U87 and U118 cells were cultured in Eagle’s Minimum Essential Medium containing 1% GlutaMAX, 100 U/ml penicillin/streptomycin, and 10% FBS (all from Thermo Fisher Scientific). HBEC cells were cultured in F-12K Medium (ATCC 30-2004), 10% FBS, 0.1 mg/ml heparin, and 30 µg/ml ECGS. All cells were cultured at 37°C in a humidified 5% CO_2_ incubator.

### Cell transduction

TRAF3IP2 was silenced (knockdown or KD) in U87 and U118 as described previously (24) by lentiviral transduction of TRAF3IP2-specific shRNA (moi1) (U87_TRAF3IP2KD_, U118_TRAF3IP2KD_). Scrambled-shRNA (U87_control shRNA_) served as the control. Polybrene^®^, a cationic polymer (Santa Cruz Biotechnology, Inc.) was used to increase transduction efficiency. Neither shRNA nor Polybrene^®^ affected cell viability and had no off-target effects (data not shown). The transduced population was selected using puromycin (500 ng/ml; Thermo Fisher Scientific), cloned at a single-cell level, and the resulting colonies analyzed for TRAF3IP2 expression by RT-qPCR. The colony displaying the highest knockdown was selected and used in the present study.

### Collection of conditioned media

Conditioned media (CM) were prepared by plating GBM cells (U87_TRAF3IP2KD_, U118_TRAF3IP2KD_ and U87_control shRNA_, U118_control shRNA_) at a density of 1,000 cells/cm^2^. After 24 h in culture, serum-free media were added. Following 24 h, conditioned media were collected and normalized to total protein before application.

### *In vitro* capillary-like tube formation assay

The tube formation ability of HBECs was assessed using Matrigel^®^ Growth Factor Reduced Basement Membrane Matrix (BD Biosciences). For this purpose, 2 × 10^4^ cells/well were seeded onto the plated Matrigel separately in six different media: endothelial basal media (EBM) (ATCC), conditioned media from U87_TRAF3IP2KD_ or U118_TRAF3IP2KD_ cultures (U87_TRAF3IP2KD_ CM or U118_TRAF3IP2KD_ CM, respectively), and conditioned media from U87_control shRNA_ or U118_control shRNA_ cultures (U87_control shRNA_ CM or U118_control shRNA_ CM, respectively) in the absence and presence of VEGF-A (40 ng/ml). The analysis was conducted 3 h after cell seeding using confocal microscopy (Nikon). The parameters of the tube formation including total loops, total tube length, and total branching points per image were assessed applying Wimasis Image Analysis Service.

### Transcriptome study using microarray analysis and panther pathway classification systems

Affymetrix^®^ gene expression microarrays were performed to obtain a differentially expressed gene list between the U87_TRAF3IP2KD_ and U87_control shRNA_ cells. Human Gene 2.0 ST CEL files were normalized to produce gene-level expression values using the implementation of Robust Multiarray Average (RMA) in the affy package (version 1.36.1) (58) included in the Bioconductor software suite (version 2.12) (59) and an Entrez Gene-specific probe set mapping (17.0.0) from the Brain array, University of Michigan (60). Array quality was assessed by computing the relative log expression (RLE) and normalized unscaled standard error (NUSE) using the affyPLM package (version 1.34.0) (61). Principal component analysis (PCA) was performed using the prcomp R function with expression values that had been normalized across all samples to a mean of zero and a standard deviation of one. Differential expression was assessed using the moderated (empirical Bayesian) t test implemented in the limma (Linear Models for Microarray and RNA-Seq) data package (version 3.14.4) (i.e., creating simple linear models with lmFit, followed by empirical Bayesian adjustment with eBayes). Analyses of variance were performed using the f.pvalue function in the sva package (version 3.4.0). Correction for multiple-hypothesis testing was accomplished using the Benjamini–Hochberg false discovery rate (FDR) (62). To perform these comparisons, probe sets whose target was not detected in any sample were eliminated from the data matrix. These criteria ensured that only those genes whose were not only highly differential between experiments but which were also expressed in a statistically significant manner were selected. Genes that exhibited highly inconsistent expression patterns, as well as genes that did not exhibit any change in expression between different cell types, were excluded from the data matrix. IPA^®^ data analyses contain five modules: canonical pathway analysis and molecular network analysis, HIF-1a signaling, tumor microenvironment signaling, and neuroinflammation pathways.

### Assessment of the VEGF-A concentration in conditioned media using ELISA

Following normalization of conditioned media to total protein, the secreted VEGF levels in conditioned media of U87_TRAF3IP2KD_, U87_control shRNA_, U118_TRAF3IP2KD_, and U118_control shRNA_ were analyzed by Human VEGF ELISA Kit (R&D Systems) (n = 3 independent triplicates of condition media).

### *In vivo* brain xenograft model

All animal protocols were approved by the Animal Care and Use Committee at the Tulane University School of Medicine in New Orleans, LA, and conformed to the *Guide for the Care and Use of Laboratory Animals*, published by the National Institutes of Health (DRR/National Institutes of Health, 1996). The tumors were generated by stereotactic injection of 3 × 10^5^ U87_TRAF3IP2KD_ or U118_TRAF3IP2KD_ cells in 3 µl PBS into the left somatosensory cortex of immunodeficient NOD mice. Scrambled shRNA served as a control (Control shRNA). The U87_control shRNA_ and U118_control shRNA_ cells were similarly injected into a different cohort of NOD mice and served as controls (n = 10 mice per group). The animals were euthanized on day 45 post-tumor induction for further analysis.

### Immunohistochemistry analysis on mouse GBM tumor

The tumor tissues were dissected, fixed in 10% neutral buffered-formalin, embedded in paraffin, sectioned at 5-μm thickness, and used for IHC according to standard protocols. The tissue sections were analyzed using primary antibodies against TRAF3IP2 (Santa Cruz Biotechnology, Inc.), IL-8, Ki67, or caspase 3 (Abcam). The sections were imaged using a ZEISS Axioscope microscope.

### Western blotting

M-PER Mammalian Protein Extraction Reagent (Cat#78503, Thermo Fisher Scientific, Waltham, MA) and Proteinase Inhibitor Cocktail (Cat#P8340, Sigma-Aldrich, St. Louis, MO) were used to extract proteins from U87_TRAF3IP2KD_, U87_Control shRNA_, U118_TRAF3IP2KD_, and U118_Control shRNA_ cells. After gel electrophoresis of equal amounts of protein using 12% Precise Tris–Glycine Gels (Cat#0025267, Thermo Fisher Scientific), Laemmli sample buffer (Cat#161-0747, Bio-Rad Laboratories, Hercules, CA), and BenchMark Pre-Stained Protein Ladder (Cat#10748-010, Invitrogen, Carlsbad, CA), the proteins were electroblotted and the following primary antibodies were used: GAPDH (0.0002 mg/ml; Cat#ab9485, Abcam, Cambridge, MA), TRAF3IP2 (0.01 mg/ml; Cat#WH0010758M1-100UG, Sigma-Aldrich), CD31 (0.01 mg/ml; Cat#PA5-16301, Invitrogen), IL1β (0.01 mg/ml; Cat#710331, Invitrogen), IL6 (0.01 mg/ml; Cat# MA5-23698, Invitrogen), IL8 (0.01 mg/ml; Cat# PA5-86028, Invitrogen). Goat Anti-Rabbit IgG-HRP (Cat#sc-2004, Santa Cruz Biotechnology, Inc.) or Donkey Anti-Mouse IgG-HRP (Cat#sc-2318, Santa Cruz Biotechnology, Inc.) served as secondary antibodies.

### Data mining

Data from the Ivy Glioblastoma Atlas Project were accessed at https://glioblastoma.alleninstitute.org. Utilizing the RNA-Seq feature, data were queried for TRAF3IP2 expression to yield differential expression data based on tumor regions. Unfiltered data were normalized for log2 intensity of expression level, downloaded, and analyzed by ANOVA followed by Tukey’s posttest using GraphPad Prism.

Clinical tumor data are from The Cancer Genome Atlas (TCGA), and normal controls are from Genotype-Tissue Expression (GTEx) accessed through GEPIA (http://gepia.cancer-pku.cn). For forest plots and survival studies, the group cutoff for high expression vs. low expression of the gene of interest is the median. The hazard ratio (HR) is calculated based on the COX proportional hazards model. Hazard ratios are for the “high expression” group for each gene and refer to overall survival (OS) or disease-free survival (DFS).

### Statistical analysis

The data were analyzed using Prism GraphPad 6 software (GraphPad Software, Inc.). Two-sided, unpaired t-test and one-way ANOVA were used to analyze the data for significance. Kaplan–Meier survival plots were created. All experiments were performed in triplicate. *p* < 0.05 was considered significant. All microarray analyses were performed using the R environment for statistical computing (version 2.15.1).

## Data availability statement

The raw data supporting the conclusions of this article will be made available by the authors, without undue reservation.

## Ethics statement

The animal study was reviewed and approved by Animal Care and Use Committee at the Tulane University School of Medicine in New Orleans, LA, and conformed to the Guide for the Care and Use of Laboratory Animals, published by the National Institutes of Health (DRR/National Institutes of Health, 1996).

## Author contributions

AI, FD, ZB, and KW executed this study. RM, SB, and AD performed *in vitro* assays. RI and EA provided the rationale and design for the study. RI, EA, BC, and AI wrote the manuscript. All authors read and approved the final manuscript.

## Funding

This project is supported through grants from the Alliance of Cardiovascular Researchers (to AI, KW, RI and to EA). Additionally, RI received a grant for this study from the Elsa U. Pardee Foundation. Also, this work was supported in part by a grant from the National Institutes of Health (P30GM145498) to RI. BC is a recipient of the Department of Veterans Affairs Research Career Scientist award (BX004016) and is supported by the U.S. Department of Veterans Affairs, Office of Research and Development-Biomedical Laboratory Research and Development (ORD-BLRD) Service Award VA-I01-BX004220.

## Acknowledgments

The authors would like to specially thank and express their gratitude to Hamid Izadpanah for the graphical works in **Figure 5**, and the Department of Pathology and Cancer Center at Tulane for histology and flow cytometry. We also thank Yasmine Rashad for her contribution to this manuscript.

## Conflict of interest

The authors declare that the research was conducted in the absence of any commercial or financial relationships that could be construed as a potential conflict of interest.

## Publisher’s note

All claims expressed in this article are solely those of the authors and do not necessarily represent those of their affiliated organizations, or those of the publisher, the editors and the reviewers. Any product that may be evaluated in this article, or claim that may be made by its manufacturer, is not guaranteed or endorsed by the publisher.

## Supplementary material

Supplementary Figure 1–3 Kaplan Meier Plots demonstrating the effect of gene expression on overall survival (OS) or disease-free survival (DFS) in GBM. Data is from The Cancer Genome Atlas (TCGA), accessed through GEPIA (http://gepia.cancer-pku.cn). Group cutoff for high expression *vs.* low expression of the gene of interest is the median. Hazard ratio (HR) is calculated based on COX proportional hazards Model. Hazard ratios are for the “high expression” group for each gene, and refer to overall survival (OS) or disease-free survival (DFS). The presented hazard ratios and their respective 95% confidence intervals are shown as forest plots in **Figure 6**.

## Abbreviations

GBM, glioblastoma multiforme; TMZ, temozolomide; PFS, progression-free survival; OS, overall survival; DFS, disease-free survival; EPC, endothelial precursor cell; TME, tumor microenvironment; IL-17R, IL-17 receptor; HBEC, primary human brain epithelial cells; CM, conditioned media; U87_TRAF3IP2KD_, TRAF3IP2 was silenced U87; U118_TRAF3IP2KD_, TRAF3IP2 was silenced U118; U87_control shRNA_, U87 Scrambled-shRNA; U118_control shRNA_, U118 Scrambled-shRNA; EBM, Endothelial Basal Media; U87_TRAF3IP2KD_ CM, conditioned media from U87_TRAF3IP2KD;_ U118_TRAF3IP2KD_ CM, conditioned media from U118_TRAF3IP2KD;_ U87_control shRNA_ CM, conditioned media from U87_control shRNA;_ U118_control shRNA_ CM, conditioned media from U118_control shRNA;_ RMA, robust multiarray average_;_ RLE, relative log expression; PCA, principal component analysis; FDR, false discovery rate; TCGA, The Cancer Genome Atlas; GTEx, Genotype-Tissue Expression; HR, hazard ratio; SSCx, somatosensory cortex; VE-cadherin, vascular endothelial cadherin; ANGPT2, angiopoietin 2.
