# Supplementary figures and images for: Targeting TRAF3IP2 inhibits angiogenesis in glioblastoma
Source: Front Oncol. 2022 Aug 15;12:893820. doi: 10.3389/fonc.2022.893820 (PMC9421153; doi:10.3389/fonc.2022.893820)

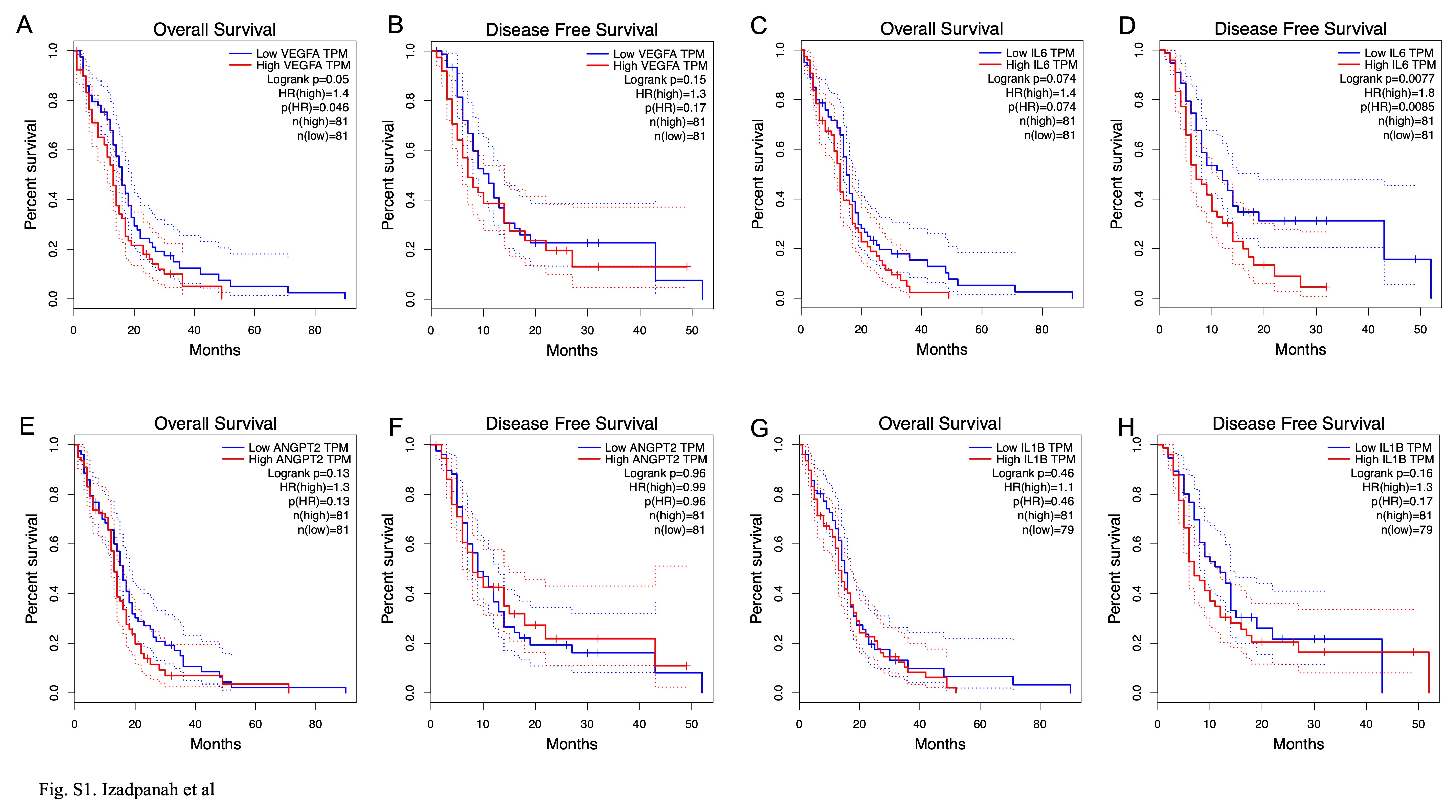

Supplement: Supplementary Figure 1–3 — Kaplan Meier Plots demonstrating the effect of gene expression on overall survival (OS) or disease-free survival (DFS) in GBM. Data is from The Cancer Genome Atlas (TCGA), accessed through GEPIA (http://gepia.cancer-pku.cn). Group cutoff for high expression vs. low expression of the gene of interest is the median. Hazard ratio (HR) is calculated based on COX proportional hazards Model. Hazard ratios are for the “high expression” group for each gene, and refer to overall survival (OS) or disease-free survival (DFS). The presented hazard ratios and their respective 95% confidence intervals are shown as forest plots in Figure 6 . [file Image_1.jpeg]

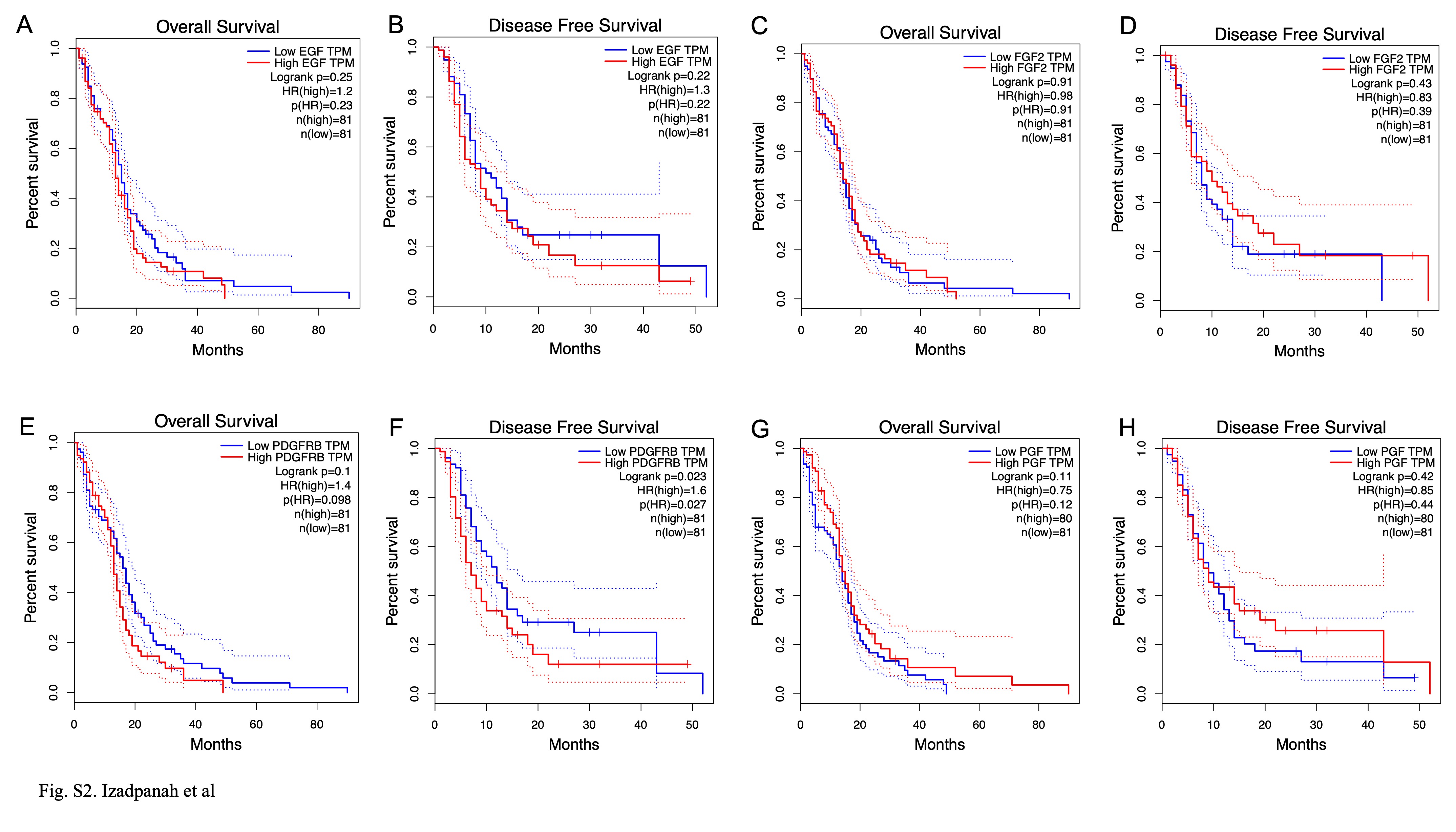

Supplement: Supplementary file 2 [file Image_2.jpeg]

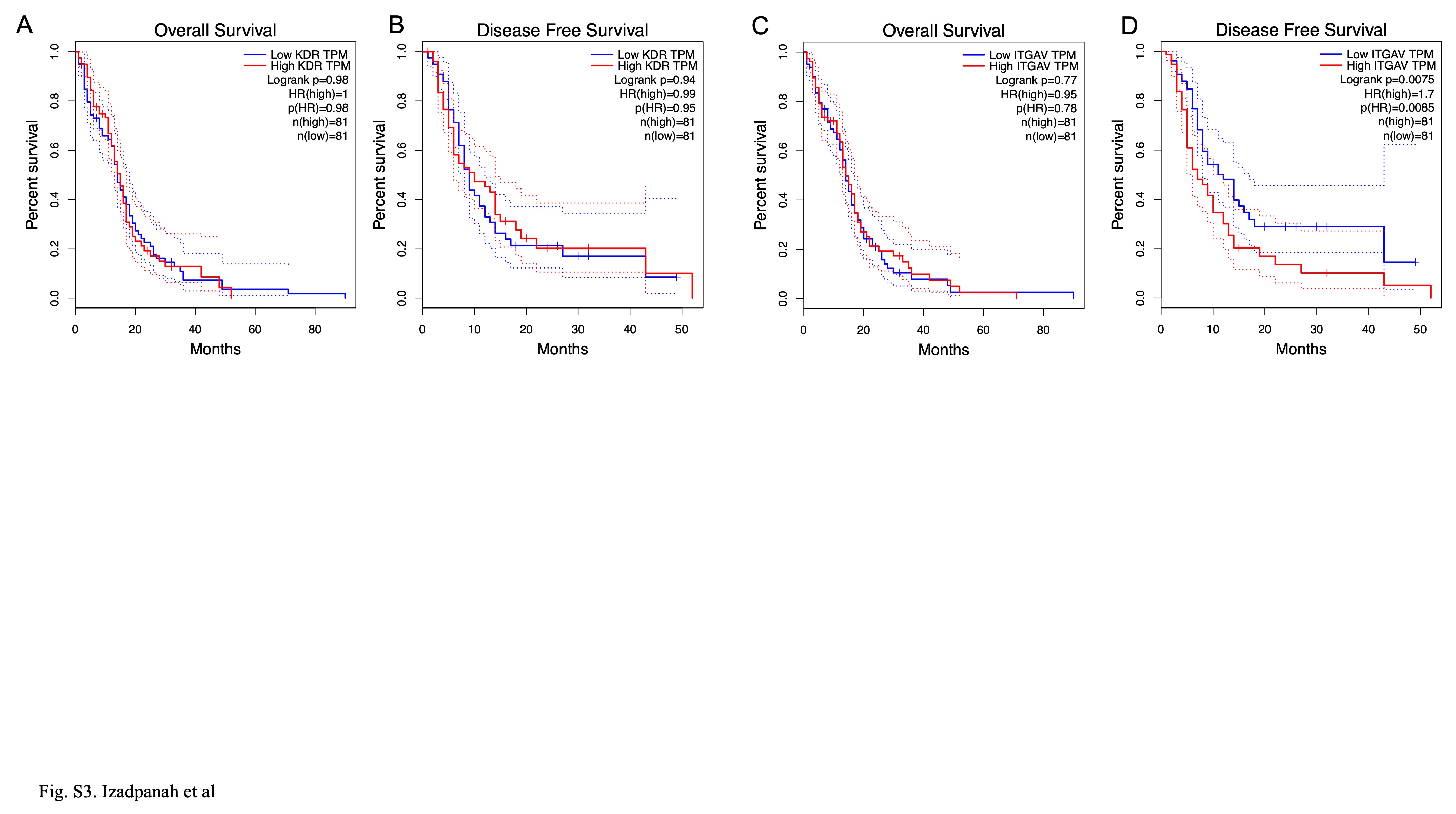

Supplement: Supplementary file 3 [file Image_3.jpeg]
